# Joint scheduling of vertical and horizontal transportation for underground container logistics in seaport terminals

**DOI:** 10.1371/journal.pone.0311536

**Published:** 2024-11-22

**Authors:** Chengji Liang, Yu Wang, Bin Lu, Yaohong Jin

**Affiliations:** 1 Logistics Research Center, Institute of Logistics Science and Engineering, Shanghai Maritime University, Shanghai, China; 2 Shanghai Municipal Engineering Design Institute (Group) Co. LTD., Shanghai, China; Southwest Jiaotong University, CHINA

## Abstract

The underground logistics system is a relatively new concept for container transportation, which is designed to reduce the congestion and pollution on the road caused by the sharply growing number of collections and distributions of containers in the port cities. This paper considers a system where some underground logistics vehicles (ULVs) are marshaled and used to transport containers between two port terminals through a deep underground tunnel. Automated guided vehicles (AGVs) are used for horizontal transportation of containers in the above-ground yard of the terminals, and yard cranes (YCs) are used to transfer the containers vertically through a shaft linking the above-ground yard and the deep underground tunnel. To guarantee the efficiency of this system, a joint scheduling problem of the YCs and the ULVs is proposed and formulated as an integer programming model to minimize the total waiting time of the YCs and ULVs. Taking marshaling and congestion of the ULVs into consideration, a Genetic Algorithm is developed to solve the problem. Numerical experimental results prove the efficiency of the proposed algorithm, and different marshaling strategies are compared. Our research provides a scientific foundation for developing underground logistics systems in large port cities.

## Introduction

Container collection and distribution of large-scale seaports mostly rely on road transportation, and the expansion of terminals leads to pressing problems in the port cities such as traffic congestion, air pollution, and the shortage of land resources [1–3]. It is hard to solve all these problems with traditional management methods based on the current collection and distribution system of the seaports. With the sharply growing throughputs of the port terminals in China, we consider a relatively new logistics system in which deep underground distribution is used as a green and efficient new mode for container transportation among different port terminals and large logistics centers in the port city. The system is based on the one proposed by Fan et al.(2020) [4] and is described in detail below.

**Fig 1:**
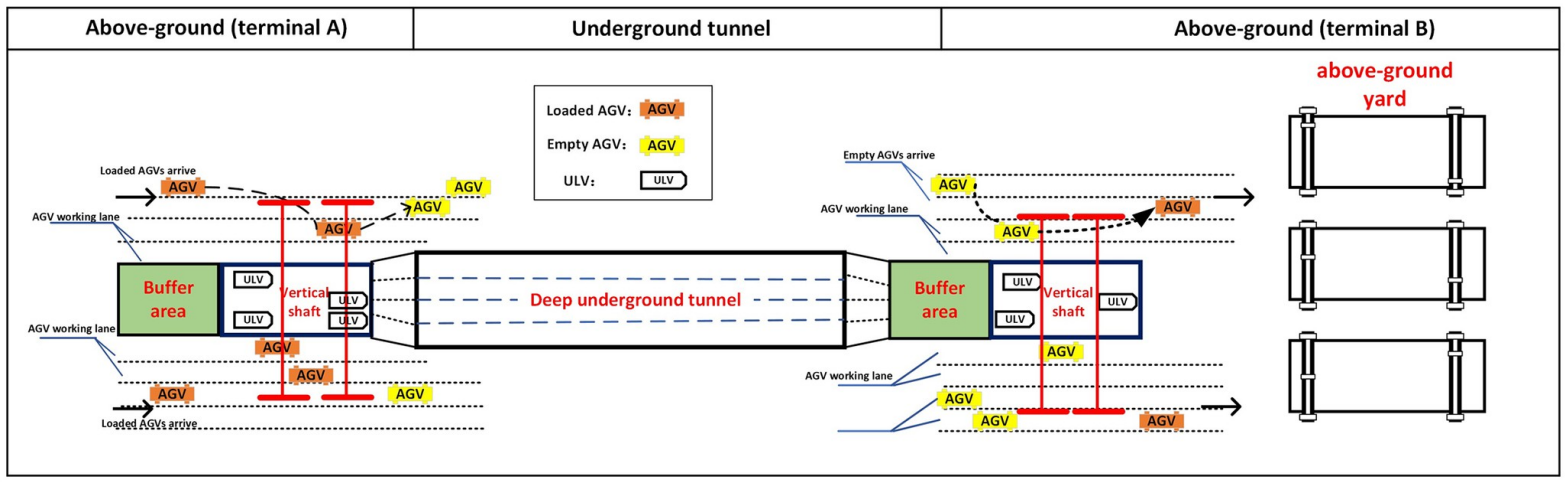
Automated container terminals with an underground logistics system.

In a general design of container terminals with an underground logistics system, as shown in Fig 1, containers are transported between terminals A and B through a deep underground tunnel. For containers from terminal A to terminal B as an example, AGVs are used to transport the containers from the above-ground yard of terminal A to a YC over the entrance of a shaft at the end of the underground tunnel. The YC is used to unload each container from the AGVs and transfer it vertically through the shaft to the ULVs in the tunnel. The AGVs will wait in a buffer area next to the shaft if the YC is busy. Each ULV can handle one container each time, and several ULVs can be marshaled to travel together. The ULVs transport the containers from terminal A to B through the deep underground tunnel, and a YC in terminal B will transfer the containers from the ULVs to the AGVs in terminal B through a similar vertical shaft at the other end of the tunnel. The containers from terminal B to A can also be handled in a reverse direction. Notice that terminal B can also be a logistics center in the city.

**Fig 2:**
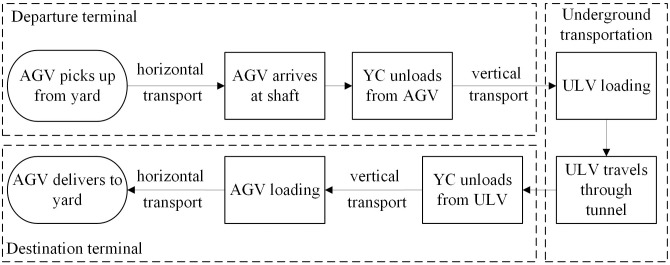
Operational process of the underground container logistics system.

Fig 2 shows the operational flow of the system considered. It is essential to optimize the scheduling of the YCs and ULVs in the system such that the efficiency of the container terminals can be improved by taking full advantage of the underground logistics system. In this paper, we consider a joint scheduling problem of the YCs at the shaft and the ULVs in the deep underground tunnel to minimize their total weighted waiting time, and the marshaling policy and the congestion of the ULVs are considered.

The research on underground logistics systems (ULS) is in a blooming stage [5]. Early research on ULS dates back to the 1880s conducted by Japan’s Engineering Research Institute. In mid-19th century, Britain established an urban pipeline postal system powered by rapid gas flow. The experts in the Netherlands also began research on ULS in the 1990s, the most famous of which was flower transportation at Amsterdam Airport [6]. In the 20th century, the United States began to build ULS and established a pipeline cabin logistics system research center [7]. More recently, Germany has vigorously studied the three-dimensional transportation system of future cities, which has the advantages of low cost and low energy consumption [8]. The application of ULS have attracted interests to large port cities in China, since the growing throughputs of the ports lead to lots of conflicts in the development of the port and the city such as road congestion and air pollution [9–11].

The improvements of tunnel excavation technology and automated equipment give the essential basis of deep underground exploitation, which facilitates the studies on the feasibility of underground container transportation. Henry Liu et al.(2004) [12] considered that the pneumatic capsule pipeline (PCP) can be used to collect and distribute containers in the New York Port and the adjacent New Jersey Port to the amount of 7.6 million TEUs per year. Stein et al.(2005) [13] studied the feasibility of using the Cargo-Cap system for container transportation between ports and inland, which has considered marshaling and has an efficiency of 136TEU/h. Vernimmena et al.(2007) [14] studied the feasibility of using the underground container transportation system for collection and distribution in the port of Antwerp, and designed a solution to underground container transportation between the two banks of the Scheldt River. Chen et al.(2018) [15] used the Yangshan Port in Shanghai, China, as an example to assess whether an underground cargo transport system could reduce the cost, time, etc., of above-ground freight container transport.

Recently, the scheduling problems at container terminals involving different equipment have been widely studied addressing multiple perspectives [16–24], but only a handful of studies have considered a joint scheduling with underground container logistics system. Regarding the connection between underground container logistics and the above-ground yard, Gao Y. et al.(2019) [25] considered an underground container logistics system with an underground parking garage with a buffer zone for loading and unloading operations. They proposed a mixed integer nonlinear programming model to minimize the waiting time of the ULVs. Pan Y et al.(2019) [26] established a two-stage model to realize road-underground-ocean container multi-modal transportation. The first stage is to determine the layout, and the second stage is a simulation model on the amount of cargo that can be transported within a given time. Fan Y. et al.(2020) [4] suggested establishing a connection station to connect underground logistics with the port yard. They proposed an optimization model to deal with the uncertainty of over-limit flow, and proved through case analysis that the connection station can alleviate traffic congestion. Gao et al.(2023) [27] considered an underground container logistics system in which yard trucks serve different handling points and yard cranes perform both loading and unloading. They described and solved a mixed integer programming problem using an adaptive genetic algorithm to schedule the yard trucks to minimise the total time for handling containers in an underground container logistics system. Liang et al.(2022) [28] integrated the scheduling problem and the configuration problem for ULVs and AGVs and developed a mathematical model to minimise the maximum completion time of the shafts.

Even though there are a handful of papers that considered scheduling problems involving underground container logistics, none of them considered the joint scheduling of yard cranes at the shaft and the ULVs in the deep underground tunnel, and none of them considered the marshaling policy of the ULVs. In this paper, we propose a joint scheduling problem for the YCs and the ULVs to minimize the total weighted waiting time and take marshaling policy and congestion into consideration.

The remaining of the paper is organized as follows: the next section describes the joint scheduling problem precisely, and the problem is formulated as an integer programming model; the Genetic Algorithm Design section proposes a Genetic Algorithm to solve the problem efficiently; the Experiments and Discussions section proposes the design of the experiments and the results; the last section concludes the paper.

## Problem description and formulation

**Fig 3:**
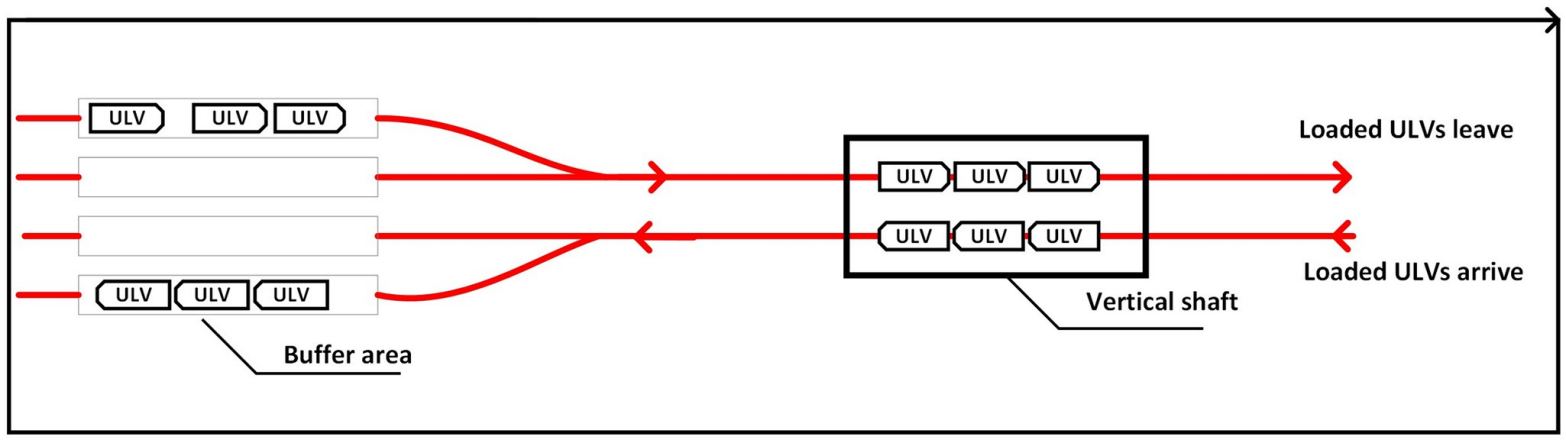
Underground operations with a buffer area.

Based on two automated container terminals with an deep underground tunnel as shown in Fig 1, the joint scheduling problem can be described precisely as follows. There is a set of containers stored in the above-ground yard of a departure terminal, denoted as D. A set of AGVs will pick up the containers and deliver them to the entrance of a vertical shaft linking the above ground yard and a deep underground tunnel. There is a set of cranes at the shaft, denoted as K, and the cranes would load the containers from the AGVs to the ULVs in the underground tunnel. Fig 3 illustrates a general case of the underground operations, where the ULVs are considered to be marshaled by a given policy such that a given number of ULVs always travel together at a given frequency. If all the cranes at either of the shafts are occupied, then new coming ULVs have to wait in a buffer area until the shaft and the cranes are free. The ULVs transport the loaded containers to a destination terminal, where there is also a vertical shaft at the end of the tunnel and equipped with a set of cranes, denoted as G. The cranes at the destination terminal will unload the containers from the ULVs to the AGVs.

Without of loss of generality, we have the following assumptions:

(1) The AGVs carrying containers arrive at the shaft of the departure terminal at a given frequency. The AGVs at the destination terminal are sufficient such that the cranes at the shaft do not need to wait for the AGVs after transferring the containers from the ULVs to the above ground.
(2) All containers are 40 feet, each yard crane at the shaft handles at most one container at a time, and the time of a single operation is known.
(3) The one-trip transportation time of the ULV in the underground tunnel is known.

With the sets and notations defined below, the joint scheduling problem of the cranes at the shaft and ULVs can be formulated as an integer programming model (IP).

*Sets and Indexes*:

D Set of containers to be handled at the departure terminal.

K Set of cranes at the departure terminal.

G Set of cranes at the destination terminal.

V Set of marshaled ULV groups.

*T* Processing time of handling one container by the yard crane at the shaft.

*L* Single-trip traveling time of the ULVs in the tunnel.

*c* Number of marshalled vehicles in each ULV group.

*u* The departure frequency of the ULVs.

*M* A sufficiently large constant.

*t*_*i*_ The arrival time of the AGV carried container *i*, i∈D.

*l*_*v*_ *l*_*v*_ = *u*(*v* − 1) is the planning time of marshaled ULV group v∈V arriving at. the shaft of the departure terminal.

*Variables*

*x*_*ikv*_ *x*_*ikv*_ = 1 if containers *i* is handled by crane *k* and is loaded by marshaled ULV group *v*, otherwise *x*_*ikv*_ = 0, i∈D, k∈K, v∈V.

*y*_*igv*_ *y*_*igv*_ = 1 if container *i* is transported by ULV group *v* and is handled by crane *k*, otherwise *y*_*igv*_ = 0, i∈D, g∈G, v∈V.

*z*_*ijk*_ *z*_*ijk*_ = 1 if container *i* and *j* are both handled by crane *k*, and *j* is handled just after *i*, otherwise *z*_*ijk*_ = 0, *i*,j∈D, k∈K.

*w*_*ijg*_ *w*_*ijg*_ = 1 if container *i* and *j* are both handled by crane *g*, and *j* is handled just after *i*, otherwise *w*_*ijg*_ = 0, *i*, j∈D, g∈G.

*e*_*v*_ the real arrival time of the marshaled ULV group v∈V at the shaft of the departure terminal.

*s*_*ikv*_ the starting time of crane *k* unloading container *i* from AGV to the ULV group *v* at the shaft of the departure terminal, i∈D, k∈K, v∈V.

*p*_*igv*_ the real arrival time of container *i* carried by ULV group *v* at crane *g* of the destination terminal, i∈D, g∈G, v∈V.

*O*_*igv*_ the starting time of crane *g* unloading container *i* from ULV group *v* at the destination terminal, i∈D, g∈G, v∈V.

*h*_*v*_ the real leaving time of marshalled ULV group *v* at the destination terminal.

*Integer programming model* (*IP*)

$$\begin{array}{cc} \text{min}\;W= & \alpha \sum_{i\in D}\sum_{k\in K}\sum_{v\in V}x_{ikv}\left(s_{ikv}-t_{i}\right)+\beta \sum_{v\in V}\left(h_{v}-l_{v}\right)+\gamma \sum_{i\in D}\sum_{j\in D}\sum_{k\in K}z_{ijk}(\sum_{v\in V}s_{jkv}\\ & -\sum_{v\in V}s_{ikv}-T)+\gamma \sum_{i\in D}\sum_{j\in D}\sum_{g\in G}w_{ijg}\left(\sum_{v\in V}o_{jgv}-\sum_{v\in V}o_{igv}-T\right) \end{array}$$ (1)

$$\begin{array}{cc} s.t. & \sum_{k\in K}\sum_{v\in V}x_{ikv}=\sum_{g\in G}\sum_{v\in V}y_{igv}=1,\;\forall i\in D \end{array}$$ (2)

$$\begin{array}{c} \sum_{i\in D}\sum_{k\in K}x_{ikv}=\sum_{i\in D}\sum_{g\in G}y_{igv}=c,\;\forall v\in V \end{array}$$ (3)

$$\begin{array}{c} \sum_{i\in D}\sum_{k\in K}z_{ijk}\leq 1,\;\forall j\in D \end{array}$$ (4)

$$\begin{array}{c} \sum_{j\in D}\sum_{k\in K}z_{ijk}\leq 1,\;\forall i\in D \end{array}$$ (5)

$$\begin{array}{c} \sum_{i\in D}\sum_{g\in G}w_{ijg}\leq 1,\;\forall j\in D \end{array}$$ (6)

$$\begin{array}{c} \sum_{j\in D}\sum_{g\in G}w_{ijg}\leq 1,\;\forall i\in D \end{array}$$ (7)

$$\begin{array}{c} s_{ikv}-t_{i}\geq M\left(x_{ikv}-1\right),\;\forall i\in D,k\in K,v\in V \end{array}$$ (8)

$$\begin{array}{c} s_{ikv}-l_{v}\geq M\left(x_{ikv}-1\right),\;\forall i\in D,k\in K,v\in V \end{array}$$ (9)

$$\begin{array}{c} s_{jkv}-s_{ikv}-T\geq M\left(z_{ijk}-1\right),\;\forall i,j\in D,k\in K,v\in V \end{array}$$ (10)

$$\begin{array}{c} e_{v}-\left(s_{ikv}+T\right)\geq M\left(x_{ikv}-1\right),\;\forall i\in D,k\in K,v\in V \end{array}$$ (11)

$$\begin{array}{c} p_{igv}=\left(e_{v}+L\right)\cdot y_{igv},\;\forall i\in D,g\in G,v\in V \end{array}$$ (12)

$$\begin{array}{c} O_{igv}-\left(p_{igv}+T\right)\geq M\left(y_{igv}-1\right),\;\forall i\in D,g\in G,v\in V \end{array}$$ (13)

$$\begin{array}{c} O_{jgv}-O_{igv}-T\geq M\left(w_{ijg}-1\right),\;\forall i,j\in D,g\in G,v\in V \end{array}$$ (14)

$$\begin{array}{c} h_{v}-\left(o_{igv}+T\right)\geq M\left(y_{igv}-1\right),\;\forall i\in D,g\in G,v\in V \end{array}$$ (15)

*x*_*ikv*_, *y*_*igv*_, *z*_*ijk*_, *w*_*ijg*_ are binary, *e*_*v*_, *s*_*ikv*_, *p*_*igv*_, *O*_*igv*_ and *h*_*v*_ are nonnegative integers, ∀*i*, j∈D, k∈K, g∈G, v∈V.

The objective (1) is to minimize the total weighted waiting time of the yard cranes at the shaft, the AGVs, and the marshaled ULVs in the underground tunnel, where *α*, *β*, and *γ* are the corresponding weight parameters. As in practice, we consider the cranes to have higher priority so that *α* > *β* > *γ*. Constraint (2) indicates that each container can only be operated by one yard crane; Constraint (3) indicates that the marshalled ULV group must be fully loaded before it can leave for transportation; Constraints (4) to (7) indicate that each crane has one and only one job before and after the incumbent one; Constraint (8) indicates that the crane at the departure terminal must wait for the AGVs carrying the container to arrive before it can operate; Constraint (9) represents the crane at the departure terminal can only be loaded and unloaded after the underground ULV marshalling arrives at the shaft mouth; Constraint (10) is expressed as the time series of loading and unloading events of each crane at the departure terminal; Constraint (11) indicates that the marshalled ULVs must departure after all loading and unloading operations finished; Constraint (12) is the time that container i carried by marshalled ULV group v arriving at crane k in the destination terminal; Constraint (13) means that the crane at the destination terminal must wait for the arrival of the loaded marshalled ULV group v before starting the loading and unloading operations; Constraint (14) is the time sequence of loading and unloading operations of each crane at the destination terminal; Constraint (15) indicates that the marshalled ULVs must wait for all corresponding containers to be loaded and unloaded to the AGVs before leaving.

## Genetic algorithm design

The joint scheduling problem of cranes at the shaft and the marshaled ULVs is obviously NP-hard. As most NP-hard problems, it is hard and inefficient to pursuing the optimal solution for real problems. A Genetic Algorithm (GA) is proposed in this section to solve the proposed problem efficiently. Genetic algorithm is a classic population-based evolutionary algorithm. It is a meta-heuristic derived from natural selection and evolution theory. GA has shown outstanding performances in solving large-scale sequencing and scheduling optimization problems, as the one proposed in this paper, in terms of global search capability and calculation efficiency.

### Chromosome encoding

**Fig 4:**
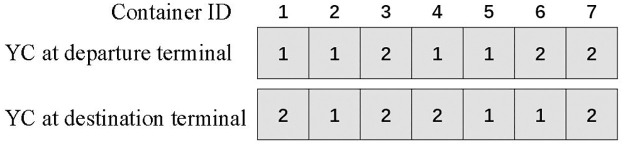
Example of chromosome encoding.

An integer encoding is considered for the joint scheduling problem as follows. In each generation, there is a set of individuals each of which is a chromosome. The chromosome length is twice the number of the containers. Namely, if the number of containers is *n*, each individual has 2*n* genes, and the value *k* in the *i*-th gene indicates that container *i* is handled by crane k∈K at the departure terminal. For gene *n* + 1 to 2*n*, the value *g* at the (*i* + *n*)-th gene indicates that the container *i* is handled by crane g∈G at the destination terminal. As shown in Fig 4, there is an example of the individuals in which 7 containers are considered, and there are 14 genes shown in two lines. Container 1, for example, is operated by crane 1 at the departure terminal, transported to the shaft by marshaled ULVs, and then is handled by crane 2 at the destination terminal.

### Fitness and selection method

In this paper, the reciprocal of the objective function (1) is used as the fitness value, namely *Fitness* = 1/*W*. It can not only contribute to a quick convergence, but also can ensure that the algorithm will pursue the global optimal solution to a large extent. The classic roulette method is used for the selection operation in each generation to ensure a constant population scale, where the individuals with better fitness have larger probabilities to be selected during each generation.

### Crossover and mutation

**Fig 5:**
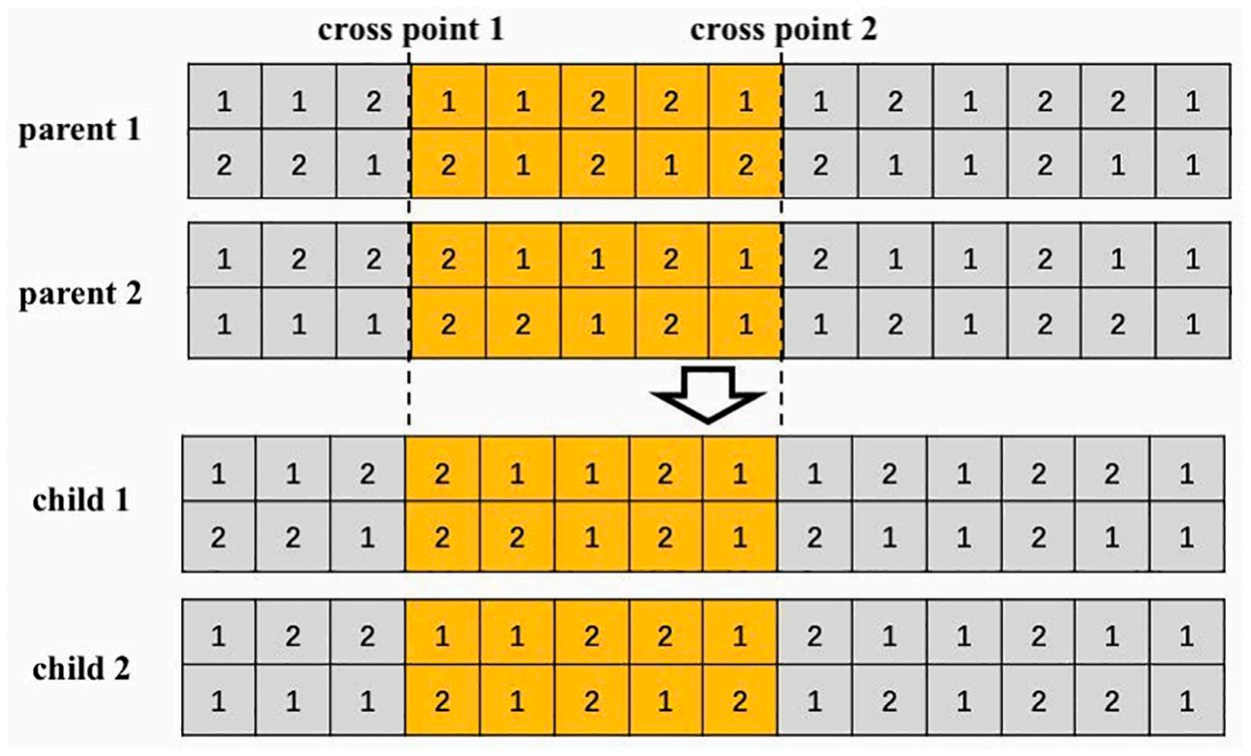
Example of crossover strategy.

In each iteration, new individuals are generated by crossover and mutation operations for each pair of selected chromosome in current generation. As shown in Fig 5, a two-point crossover strategy is used in this algorithm. For each pair of individuals in the incumbent generation, they will take the crossover strategy following a predetermined crossover probability. The crossover operator will randomly generate two indexes of the genes, and the selected individuals will exchange the genes between the generated indexes. Two new individual will be generated after the exchange.

Mutation operation takes place after every pair of individuals has been checked and handled based on the crossover operator. For each new generated individual, two indexes of the genes will be randomly generated according to a predetermined mutation probability. The values at these two genes will be exchanged to obtain a new individual if the two indexes are generated.

### Overall process of GA

**Fig 6:**
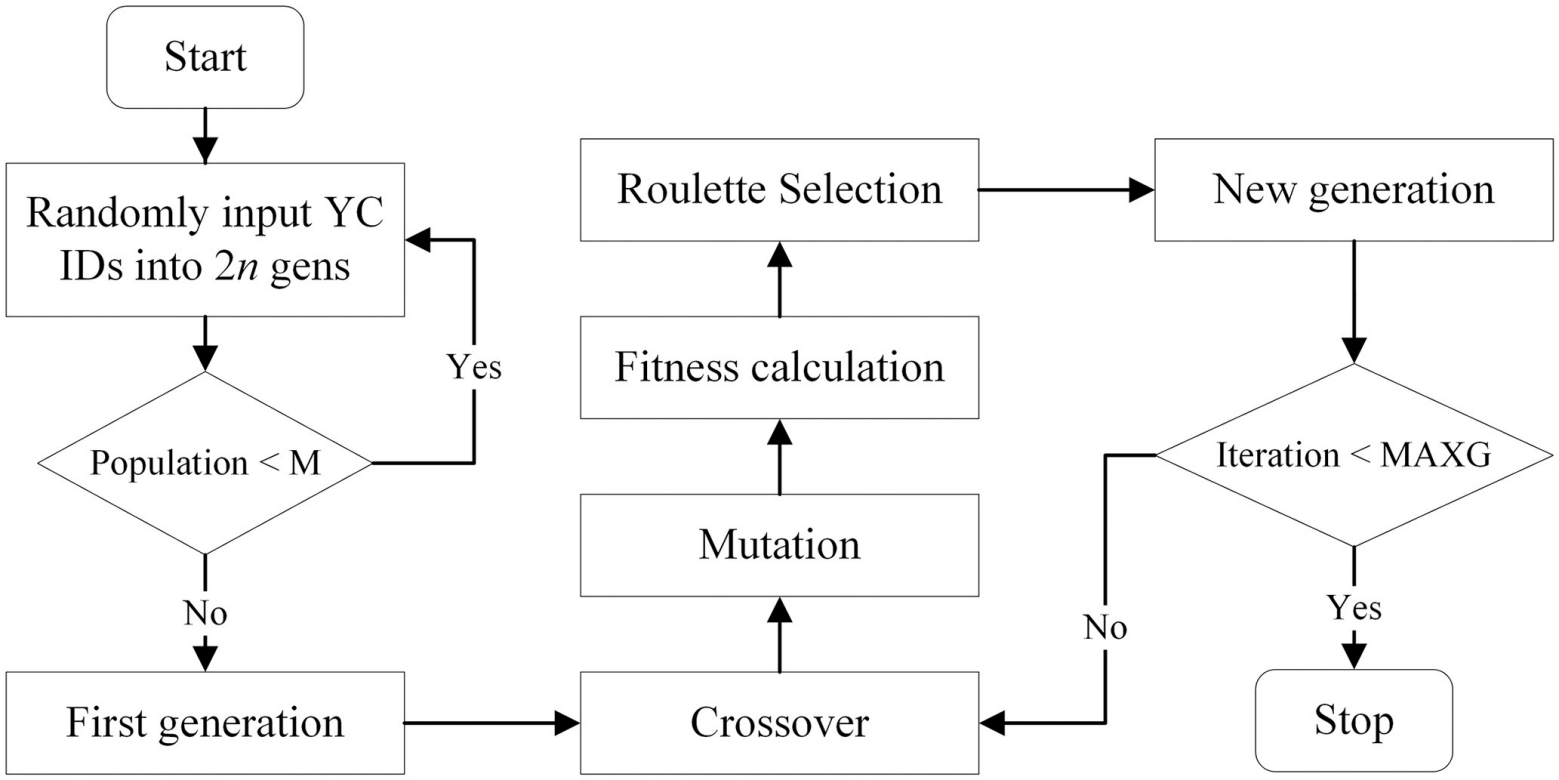
Flowchart of the GA algorithm.

While applying the GA algorithm, we first generate an initial group of individual chromosomes as the first generation. Let *M* be the given population size of the first generation. Based on the chromosome encoding rules, the ID of YCs in the departure and destination terminals are randomly selected as the 1 to *n* gens and *n* + 1 to 2*n* gens for each individual, respectively, and this random generation process repeated for *M* times to gain the first generation. Starting from the first generation, in each iteration of GA, the crossover and mutation process are conducted for each pair of individuals to generate new individuals. The fitness is then calculated for all the individuals and the roulette selection process is conducted to keep *M* individuals as the new generation. The GA process iterated until a sufficient number of generations are checked, denoted as MAXG. Fig 6 shows the flow of the GA algorithm.

## Experiments and discussions

In this section, we first validate the proposed model and the GA algorithm based on two small-scale instances. Experiments based on large-scale instances are then conducted to show the efficiency of the proposed algorithm on solving practical size problems. Finally, sensitivity analysis are tested on different departure frequencies of the ULVs.

### Results on small-scale instances

To show the validation and efficiency of our algorithm, we first generate two small-scale instances with 20 containers, and compare the performance of the proposed GA algorithm to solving the IP model directly by a commercial solver, GAMS. The algorithm is programmed in MATLAB. Related parameters in the instances are defined based on investigation with port equipment suppliers, which are described below along with the parameters in the GA algorithm.

There are 2-yard cranes at departure and destination terminals, respectively, and 5 marshaled ULV groups in the underground tunnel with a departure frequency of 7 minutes. It takes 2.5 minutes for the crane at each shaft to lift and load/unload one container, and it takes 10 minutes for the marshaled ULV group to travel through the underground tunnel. Let *α* = 5, *β* = 0.5 and *γ* = 1 in the objective function. The arrival moments of the AGVs at the departure terminal are generated randomly in a give range. The maximum number of iterations of GA is limited to 500 as the termination condition for this small-scale instance, the number of populations is set to be 50, and the crossover probability and mutation probability is 0.7 and 0.4, respectively.

**Fig 7:**
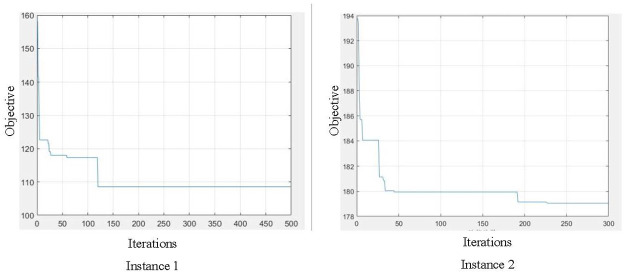
Convergence of GA.

Table 1 and Fig 7 show the results based on the small-scale instances. As can be seen from the results, the proposed GA algorithm can generate near-optimal solutions within a relatively short time. The proposed GA algorithm converged in about 150 generations for the testing instance. The difference between the objective function value generated by the algorithm and the optimal one generated by GAMS is less than 1%, while the computational time reduced a lot compared to the solver.

**Table 1:** Results of different methods based on small-scale instances.

	Computational time (sec)			Objective function		
	GAMS	GA	gap	GAMS	GA	gap
instance 1	117	101	13.68%	108.15	108.55	0.37%
instance 2	277	101	63.54%	177.35	179.05	0.96%

### Results on large-scale instances

For problems in real practice, there could be more containers and more equipment. We then generate random instances of practical-scale to evaluate the efficiency of the proposed algorithm. For a randomly generated instance with 100 containers, and 5 marshaled ULV groups, we solve the problem by the proposed GA algorithm with a departure frequency equal to 7 minutes times a floating rate of up to 20%. Other parameters are the same with those in small-scale tests. As shown in Table 2, the results of a fixed 7-minute frequency are compared to the ones with a floating rate.

**Table 2:** Results of fixed and floating rate on ULV departure frequency.

Methods	Waiting time (min)		
	Cranes	AGVs	ULVs
**Floating**	31.40	759.30	302.00
**Fixed**	39.80	807.50	318.60

Based on the results shown in Table 2, for the case considering a floating rate on the departure frequency of ULVs, the total waiting time of cranes, AGVs, and ULVs is all shorter than that of the case with a fixed frequency. The weighted sum of the total waiting time is reduced by 7.47% while taking advantage of the changing frequency for ULVs. It shows that considering a flexible departure strategy for marshaled ULV groups in the underground logistics system can contribute to improving the coordination among the vertical and horizontal transportation equipment.

### Results on different frequencies of ULVs

We then study the influence of the floating rate on the departure frequency of the ULVs. As the results shown in Table 3, a 20% floating rate is appropriate for optimizing the weighted sum of the waiting time. The best objective function value found by GA decreases with the floating rate growing from 5% to 20%, while the value is shown to be increasing when the rate grows from 20% to 30%.

**Table 3:** Results on different floating rates of departure frequency of ULVs.

Rate	Best objective value (min)	Rate	Best objective value (min)
5%	918.00	20%	838.65
10%	908.85	25%	963.14
15%	896.23	30%	1109.82

Finally, based on the results above, we consider a departure frequency of ULVs with a floating rate of 20%, and generate and solve two sets of instances with 600 containers and 1200 containers, respectively. For each set of instances, different numbers of marshaling groups and baseline departure frequencies are compared. The number of marshaled ULV groups ranges from 3 to 5, and the baseline frequency ranges from 6 to 9.

As the results are shown in Table 4, for both instances with 600 and 1200 containers, the objective takes the best value when the number of marshaling groups is 5 and the baseline frequency is 8 minutes. Under the same task scale, it is not that the number of marshaling is more. As shown by the results, for a given number of containers, the objective value is not strictly decreased with the increase of the marshaled ULV groups or the baseline frequencies. It implies that in practice it is not necessary to increase the number of ULVs or accelerate the departure frequency. The scheduling of all vertical and horizontal transportation equipment in container terminals with underground logistics systems should be considered comprehensively.

**Table 4:** Results under different marshaling numbers and departure frequency.

Containers	Marshalled ULV groups	Baseline frequency of ULVs (min)	Objective value (min)
600	3	6	76636.00
	3	7	109568.75
	3	8	141568.45
	4	7	50860.90
	4	8	76057.75
	5	8	35847.35
	5	9	56019.65
1200	3	6	283680.05
	3	7	410258.20
	3	8	536402.20
	4	7	187155.35
	4	8	282707.65
	5	8	129996.15
	5	9	206365.95

## Conclusions

This paper considers a joint scheduling problem of the marshaled ULVs in a deep underground tunnel and the yard cranes of two terminals transferring the containers from above the ground to the ULVs through vertical shafts. The problem is to minimize the total waiting time of the vertical transportation by the cranes and that of the horizontal transportation by the ULVs in the deep underground tunnel connecting two terminals in the system, and the marshaling policy and congestion of the ULVs are considered. An integer programming model is proposed for the problem, and a genetic algorithm is designed to solve the problem efficiently. Experimental results show that the proposed GA algorithm outperforms solving the IP model directly by GAMS, and the joint scheduling of vertical and horizontal transporters is essential for reducing the waiting time in the system, and marshaled ULV groups with appropriate floating departure frequency will contribute to improving the efficiency of container transportation.

Our work shows an effective and efficient way of terminal operations with underground systems. It indicates that an underground container logistics system with a deep tunnel could be used for supporting the further development of huge port cities while feasibilities and flexibilities in more aspects could be discussed. For example, one can consider the joint scheduling for more equipment at container terminals with underground logistics or take more dynamic environment features into consideration. One can also study emergency management for underground logistic systems.
